# How group-based physical and social activities preserve cognitive function in older adults

**DOI:** 10.1093/geroni/igaf122.3791

**Published:** 2025-12-31

**Authors:** Jisu Seo, Seohye Jeong, Eunna Oh, Rhayun Song

**Affiliations:** Chungnam National University, Daejeon, Republic of Korea; Chungnam National University, Daejeon, Republic of Korea; Chungnam National University, Daejeon, Republic of Korea; Chungnam National University, Daejeon, Republic of Korea

## Abstract

Both structured physical exercise and socially engaging group-based interventions have been shown to support cognitive health in older adults. This study aimed to compare the effects of Tai Chi (a physical activity) and Laughing Therapy (a social activity) to a wait-list control group on cognitive function in community-dwelling frail older adults. A three-arm randomized controlled trial was conducted over 12 weeks. Participants were randomly assigned to one of three groups: Tai Chi (n = 70), Laughing Therapy (n = 46), or a wait-list control group (n = 44). The Tai Chi group attended sessions twice a week for one hour, while the Laughing Therapy group met once a week for one hour. Cognitive function was assessed using the MoCA-K, including subdomain scores. The mean age of participants was 78.83 years (SD = 4.35). Repeated measures ANOVA, controlling for age, was used to examine group differences. After controlling for age, participants in both intervention groups showed trends of improvement across most MoCA-K domains. However, a statistically significant group difference was observed only in the language domain compared to the control group (F = 4.46, p = .013). The difference in total MoCA-K scores was not statistically significant (F = 1.20, p = .304). Group-based Tai Chi and Laughing Therapy programs demonstrated potential to enhance language-related cognitive functions among community-dwelling frail older adults. These findings underscore the importance of community-based interventions in maintaining cognitive function and highlight the need for further research to explore the underlying mechanisms and optimal strategies.

